# Development of the health literacy on social determinants of health questionnaire in Japanese adults

**DOI:** 10.1186/s12889-016-3971-3

**Published:** 2017-01-06

**Authors:** Masayoshi Matsumoto, Kazuhiro Nakayama

**Affiliations:** 1 Universal Business Solutions, 3-1-1 Kyoubashi TOKYO SQUARE GARDEN, Chuo-ku, Tokyo, 104-0031 Japan; 2 Department of Nursing Informatics, Graduate School of Nursing Science, St. Luke’s International University, Tokyo, Japan

**Keywords:** Health literacy, Social determinants of health, Health promotion

## Abstract

**Background:**

Health inequities are increasing worldwide, with mounting evidence showing that the greatest cause of which are social determinants of health. To reduce inequities, a lot of citizens need to be able to access, understand, appraise, and apply information on the social determinants; that is, they need to improve health literacy on social determinants of health. However, only a limited number of scales focus on these considerations; hence, we developed the Health Literacy on Social Determinants of Health Questionnaire (HL-SDHQ) and examined its psychometric properties.

**Methods:**

We extracted domains of the social determinants of health from “the solid facts” and related articles, operationalizing the following ten domains: “the social gradient,” “early life,” “social exclusion,” “work,” “unemployment,” “social support,” “social capital,” “addiction,” “food,” and “transport,” Next, we developed the scale items in the ten extracted domains based on the literature and included four aspects of health literacy (ability to access, understand, appraise, and apply social determinants of health-related information) in the items. We also evaluated the ease of response and content validity. The self-administered questionnaire consisted of 33 items. The reliability and construct validity were verified among 831 Japanese adults in an internet survey.

**Results:**

The scale items had high reliability with a Cronbach’s alpha of 0.92, and also adequate results were obtained for the internal consistency of the information-processing dimensions (Cronbach’s alpha values were 0.82, 0.91, 0.84, and 0.92 for accessing, understanding, appraising, and applying, respectively). The goodness of fit by confirmatory factor analysis based on the four dimensions was an acceptable value (comparative fit index = 0.901; root mean square error of approximation = 0.058). Furthermore, the bivariate relationship between HL-SDHQ and the frequency of participation in citizen’s activities was similar to the theoretical results.

**Conclusions:**

HL-SDHQ clarifies the relationship between the ten domains of the social determinants of health and health in each domain and is able to measure whether it is possible to access, understand, appraise, and apply related information. The reliability and validity of the scale were adequate.

**Electronic supplementary material:**

The online version of this article (doi:10.1186/s12889-016-3971-3) contains supplementary material, which is available to authorized users.

## Background

In recent years, although general health has improved with the development of medical and public health, health inequities continue to increase worldwide. According to final report in 2008 by the World Health Organization (WHO) Commission on the Social Determinants of Health (CSDH) [1], there is an urgent need to work toward reducing these health inequities. Differences in health conditions occur because of differences in lifestyle, environment, and medical treatment, but these are themselves determined by social, economic, and political factors.

Health differences that arise because of the society in which one is born are not the responsibility of the individual, but represent systemic gaps caused by the society. The factors that give rise to these differences have been referred to as the social determinants of health. In 1998, the WHO Regional Office for Europe announced “the solid facts” to improve awareness of the social determinants of health, which was later updated in the second edition in 2003. This publication shows ten relevant domains, organizing the evidence related to the relationship with health in a systematic manner [2]. Marmot, the editor of “the solid facts,” noted that social determinants of health were the fundamental cause of an individual’s lifestyle and so referred to them as the “cause of cause [3]”.

In the final report by the CSDH [1], it gave three proposals to help realize true health equity through social determinants. The proposals include the importance of improving health literacy in citizens, with access to information about social determinants of health, and the ability to understand, evaluate, and communicate this information with everyone.

Health literacy is defined as the ability to access, understand, appraise, and apply health information [4]. In recent years, its importance has been increasing and is considered somewhat indispensable for the realization of health. Therefore, many scales have been developed to measure health literacy, including the Test of Functional Health Literacy in Adults, the Rapid Estimate of Adult Literacy in Medicine, and the Newest Vital Sign scales, which primarily screen for patients with low health literacy in the medical field. These scales measure functional health literacy that focusses on basic reading and writing in the medical field. In addition to this type of health literacy, Nutbeam proposed other two levels of health literacy, including the higher abilities of communicative health literacy and critical health literacy [5]. The content of critical health literacy is often discussed from the point of view of public health and health promotion [6–8], and is understood as the ability to know about, and be engaged in, the social determinants of health. Thus, it is almost the same as health literacy proposed by the CSDH [1]. Similarly, Freedman et al. [9] pointed out the importance of “public health literacy” that aims at the involvement of the individual or the community in the social determinants of health. In this way, health literacy is not just about the ability to read and write health information, nor is it limited to the ability to make beneficial decisions for one’s own health, but it includes the ability to build a society in which individuals can live a healthy life and make healthy choices based on an individuals’ modifications to the social determinants of health.

At present, numerous scales do not measure health literacy with the latter meaning, and we are aware of few scientific evaluations of whether citizens can know about the social determinants of health and whether they can implement the health promotion initiatives. There are studies that are seeking to develop scales that take social determinants of health into consideration, including the All Aspects of Health Literacy Scale [10] and the European Health Literacy Survey Questionnaire (HLS-EU-Q47) [11]. The HLS-EU-Q47 includes three major domains of health—that is, health care, disease prevention, and health promotion—and the scale items under health promotion mostly consider the relationship between health and the social determinants of health. However, not all the scale items in this domain take into consideration the social determinants of health. Therefore, we developed health literacy on social determinants of health questionnaire and examined the reliability and validity.

## Methods

### Definition of Health Literacy on Social Determinants of Health

For the development of the scale, we referred to the definition of health literacy for the domain of health promotion developed by Sorensen et al. [4] because the definition integrates the concept of health literacy and social determinants of health:

*“Health literacy refers to the ability to regularly update oneself on determinants of health in the social and physical environment and derive meaning, to interpret and evaluate information on determinants of health in the social and physical environment, and the ability to make informed decisions on health determinants in the social and physical environment and also engage in joint action”.*

### Domains of the social determinants of health

Taking the definition by Sorensen et al. [4] as the starting point, we extracted nine relevant domains for the social determinants of health from the second edition of “the solid facts [2]”. This publication was chosen as our main resource material. In order to develop new scale in Japan, we had to consider the social determinants of health in Japan. Health and its major influences substantially vary with economic development but the evidence shown in the publication is also becoming clear in Japan and they are considered to be in accordance with the current conditions in Japan [12–24]. Therefore, we chose the publication as our main resource material.

“The solid facts” outlines the most important aspects of new knowledge related to social determinants of health. This includes ten domains, “the social gradient,” “stress,” “early life,” “social exclusion,” “work,” “unemployment,” “social support,” “addiction,” “food,” and “transport,” Although we considered “stress” as one of the social determinants, we did not set stress as an independent domain but included topics on stress in other domains because people are subjected to many kinds of stress in daily life. For example, in the domains on “unemployment,” “addiction,” and “work,” we included topics on stress caused by job insecurity, social settings and work.

On the other hand, we added “social capital” to the domains. The description of “social support” in “the solid facts” includes not only social support but also social cohesion, which is defined as “*the quality of social relationships and the existence of trust, mutual obligations and respect in communities or in the wider society”* [2]*.* When we considered whether to add “social cohesion” to the domain, we found that the final form of the CSDH document, “A Conceptual Framework for Action on the Social Determinants of Health, [25]” introduces social capital as well as social cohesion as important determinants to achieve health equity. Social capital is similar to social cohesion and defined as “*features of social organization, such as trust, norms, and networks that can improve the efficiency of society by facilitating coordinated actions”* [26]. Although the term, “social cohesion” was used in “the solid facts,” we added the social capital to the domain because it was widely used in the studies including European Social Survey [27]. Consequently, we operationalized these ten domains as the social determinants of health (Table 1).

**Table 1:** Social determinants of health and descriptions of each determinant

	Determinants
1.	The social gradient
	Poor social and economic circumstances affect health throughout life.
2.	Early life
	The health impact of early development and education lasts a lifetime.
3.	Social exclusion
	Being excluded from the society and treated as less than equal leads to worse health.
4.	Work
	Control over work leads to better health.
5.	Unemployment
	Job security increases health, well-being, and job satisfaction.
6.	Social support
	Less social and emotional support from others increases the likelihood of poor health.
7.	Social capital
	The existence of trust, norms, and networks in communities or in wider societies helps protect people and their health.
8.	Addiction
	Use of alcohol, drugs, and tobacco is influenced by wider social settings.
9.	Food
	Global market forces control healthy food supply.
10.	Transport
	Healthy transport means less driving and more walking and cycling and is supported by better public transport.

### Item generation

In “the solid facts,” we identified 33 items that covered the ten domains. Topics about the relationship between health and each domain were set as questionnaire items. Further, we referenced the HLS-EU-Q47 [11] to identify questions on four health literacy abilities (i.e., the abilities to access, understand, appraise, and apply information related to the social determinants of health), from among the 33 items. We identified seven items each for the accessing, understanding, and appraising abilities, and twelve items for the applying ability. All of the items were set as self-report statements. To determine the ease of response, 22 participants were asked to provide responses and opinions about items that were difficult to respond to, and items identified as being difficult to respond to were examined again, revised, and confirmed by respondents.

### Content validity

To ensure the content validity of the question items for the ten domains, the items were examined and adapted through discussions with researchers from public health, nursing, and social sciences working in related fields. No questions were revised in this task.

### Item-score

The score per question was rated 1–4 depending on the response, and the scores were summed. For example, one question was “On a scale from very easy to very difficult, how easy would you say it is to understand that the lesser the income the greater the tendency to become ill?” and were ranked on a four-point Likert-type scale (1 = very difficult, 2 = fairly difficult, 3 = fairly easy, and 4 = very easy). We also included the response “don’t know/not applicable” to help assess participants’ level of comprehension. Further, as in the HLS-EU-Q47, items with missing values were supplemented by the substitution of the average value of that item for the whole sample [28].

### Participants

Participants were recruited from registrants of an internet research service in Japan. The research company had approximately 2.5 million voluntarily registered participants at study onset, from which we sought to collect data from 1000 men and women aged 20–69 years. On October 30, 2014, potential respondents were randomly invited via email to complete the cross-sectional web-based health literacy questionnaire anonymously. Participants voluntarily signed an online informed consent form approved by our institutional review board. The present study received prior approval from the research ethics committee of St. Luke’s International University, Japan.

We sought to match the percentages of participants by gender, age group, and region (divided into eight nationwide regions in Japan) to the results of the 2010 Japanese census [29]. We accepted emailed responses from potential participants until the target number of responses was collected. From among these 1000 respondents, based on the calculation method of the HLS-EU-Q47 score, 831 people gave valid responses for ≥80% of the scale items and were targeted for analysis.

### Confirmation of scale validity

The HLS-EU-Q47 [11] comprises three sub-indices: the health care health literacy index (HC-HL), the disease prevention health literacy index (DP-HL), and the health promotion health literacy index (HP-HL). HL-SDHQ referred the definition of health literacy for the domain of health promotion; we, therefore, used the HP-HL index of the Japanese version of the HLS-EU-Q47 [30] to confirm scale validity. We expected it to better recognize health literacy which needs to realize health promotion than HP-HL index because HL-SDHQ clearly focuses on the social determinants of health. HP-HL answer categories were all phrased as “On a scale from very easy to very difficult” and ranked on a four-point Likert-type scale (1 = very difficult, 2 = fairly difficult, 3 = fairly easy, and 4 = very easy), or as “don’t know/not applicable,” which was coded as a missing value.

We also assessed the relationship between HL-SDHQ and frequency and number of participation in citizens’ activities. Nutbeam pointed out that “critical health literacy” reflects cognitive- and skill-development outcomes oriented toward supporting effective social and political action, as well as individual action. Thus, we considered that there might be a relationship between HL-SDHQ and the frequency and the number of participation in citizens’ activities including social and political action. Further, with regard to the frequency of participation in each citizens’ activity, we compared the Spearman’s correlation coefficient for the HL-SDHQ with the corresponding value for the HP-HL. We also considered that the correlation coefficient for the HL-SDHQ was higher than that for the HP-HL. This was because the HL-SDHQ, more so than the HP-HL, reflected the responses from the perspective of community health rather than individual health. With reference to the major classifications of legislation in Japan designed to promote specified non-profit activities, 25 activities were targeted, including community involvement activities. Questions were asked about the frequency of participation in each citizens’ activity, and four responses were available (4 = usually, 3 = often, 2 = sometimes, 1 = never). For the number of participation in citizen’s activity, we calculated the score based on the frequency of participation in each citizens’ activity. If the response was “usually,” “often,” or “sometimes,” we recognized the respondent with an experience of participation and gave a score of “1”. Otherwise, respondents with no experience were given a score of “0”. We summed the score of 25 activities.

We assessed the relationship between HL-SDHQ and health status. Self-rated health was measured by participants’ responses to the question, “Recently, how would you describe your state of health?” with five outcomes (5 = good, 4 = fairly good, 3 = fair, 2 = fairly poor, and 1 = poor). This was the same question and response option that appeared in a survey administered by the Ministry of Health, Labour and Welfare [31].

### Demographic and socioeconomic characteristics

The following demographic and socioeconomic characteristics were analyzed: gender, age groups, highest level of education, annual pre-tax household income in millions of yen, self-assessed living conditions, occupation, and municipality size (see Table 2). The question regarding living conditions was the same as that of a survey administered by the Ministry of Health, Labour and Welfare [31].

**Table 2:** Characteristics of study participants

	Total(*n* = 831)	
Variables	*n*	%
Gender		
Men	442	53.2
Women	389	46.8
Age in categories		
20–29	137	16.5
30–39	180	21.7
40–49	167	20.1
50–59	169	20.3
60–69	178	21.4
Age (mean ± SD)	45.5	13.5
Education attainment		
Junior high school	12	1.4
High school	188	22.6
Junior college	183	22.0
College/University	381	45.8
Graduate school	66	7.9
Others	1	0.1
Annual pre-tax household income (million yen^a^)		
< 1.5	32	3.9
1.5–3.5	146	17.6
3.5–6.0	245	29.5
6.0–8.5	141	17.0
8.5–12.5	119	14.3
≥ 12.5	32	3.9
Unknown	116	14.0
Self-assessed Living Conditions		
Very Hard	64	7.7
A little Hard	252	30.3
Common	384	46.2
A Little Well	116	14.0
Very well	15	1.8
Occupation		
Self-employed	42	5.1
Managerial and administrative	30	3.6
Professional and technical	131	15.8
Others (Routine and manual)	244	29.4
Part time	90	10.8
Homemaker	150	18.1
Student	23	2.8
Unemployed	88	10.6
Others	33	4.0
Municipality size		
Very large (population of ≥500,000)	280	33.7
Large (population of 100,000–499,999)	288	34.7
Moderate (population of <100,000)	162	19.5
Small	62	7.5
Unknown	39	4.7

^a^$1 = approximately 102 yen in Sep 2016

### Statistical analysis

#### Reliability and validity

Cronbach’s alphas were calculated to examine internal consistency. For construct validity, confirmatory factor analysis (CFA) was conducted. The number of factors was set to four, which related to the four information-processing dimensions (accessing, understanding, appraising, and applying). In the CFA, we used the comparative fit index (CFI) and the root mean square error of approximation (RMSEA) as the model fit indices. A CFI value ≥0.90 was considered to indicate acceptable model fit. For the RMSEA, a value <0.05 represented good fit, and a value <0.08 was considered acceptable [32]. Construct validity was also assessed using bivariate analysis between the HL-SDHQ, and HP-HL index of the Japanese version of HLS-EU-Q47, the frequency and the number of participating citizens’ activity and self-rated health.

We also compared the mean and standard deviation of the HL-SDHQ by demographic and socioeconomic characteristics (gender, age, education, income, living conditions, occupation, and municipality size). Data were analyzed by IBM SPSS and Amos version 23.0.

## Results

### Demographic and socioeconomic characteristics

Table 2 shows the demographic and socioeconomic characteristics of the sample. Males constituted 53.2%, and females, 46.8%; their mean age was 45.5 years. The most common educational qualification was university level, and household incomes ranged from 3.5 million to 6 million yen. Regarding self-assessed living conditions, “common” was the most common response (46.2%), followed by “a little hard” (30.3%).

The sample distribution in terms of age and income was almost in accordance with the distribution of these metrics in the Japanese population (not tabulated), but men and college/university or higher overrepresented in the studied population [29]. Regarding self-assessed living conditions, “very hard” was underrepresented in our study compared with that in a recent national study on Japanese living conditions, whereas “common,” “a little well,” and “very well” were slightly overrepresented [31].

### Scores on HL-SDHQ items

The wordings of the 33 items, and the empirical answer patterns, are displayed in Table 3. Only 2 items had higher non-response rates (Question [Q] 24 “Participate in childcare support activities” = 6.9%; Q 1 “Find out about the impact of social position on health” = 6.7%.) The percentage distributions demonstrate considerable variation in item difficulty, ranging from 1.1% (Q1) to 24.2% (Q12) for very easy, and from 7.7% (Q12) to 42.4% (Q26) for very difficult.

**Table 3:** Percentage of respondents giving each response for all HL-SDH items

Items			Very easy (%)	Fairly Easy (%)	Fairly Difficult (%)	Very difficult (%)	Don’t know/Not applicable (%)
	Dimensions/domain	On a scale from very easy to very difficult, how easy would you say it is to:…					
Q1	Access/the social gradient	Find out about the impact of social position on health	1.1	14.9	49.2	28.0	6.7
Q2	Access/early life	Find information related to the impact of the daily life of a mother on the growth of the child to be born	4.3	28.8	43.3	19.3	4.3
Q3	Access/social exclusion	Find someone who is isolated from society and whose health is failing	2.6	12.3	41.5	40.8	2.8
Q4	Access/unemployment	Find information on the relation between unemployment and stress	3.0	20.7	49.8	23.7	2.8
Q5	Access/social support	Find out the support required by someone in trouble in the community or workplace	2.9	18.5	49.9	27.1	1.6
Q6	Access/addiction	Find out smoking is not going to eliminate the cause of stress	8.4	30.2	41.4	16.1	3.9
Q7	Access/food	Find information about the relationship between dietary changes and health	9.1	40.4	38.0	11.7	0.7
Q8	Understand/the social gradient	Understand that the lesser the income the greater the tendency to become ill	7.3	31.0	45.8	11.9	3.9
Q9	Understand/early life	Understand that abuse suffered as a child has an impact even when one becomes an adult	19.3	43.4	27.0	9.9	0.5
Q10	Understand/social exclusion	Understand that being isolated from the community and workplace impacts health	15.0	41.8	33.7	8.2	1.3
Q11	Understand/work	Understand that determining how to proceed working on one’s own is related to stress	11.1	40.2	37.7	9.9	1.2
Q12	Understand/unemployment	Understand that work that is not stable becomes a huge stress	24.2	41.9	26.0	7.7	0.2
Q13	Understand/social capital	Understand that widening income disparities dilute the ties between people	14.4	37.3	35.5	10.5	2.3
Q14	Understand/addiction	Understand that in a society with a high level of stress, there is a tendency toward dependency on drugs	16.4	39.6	33.6	8.8	1.7
Q15	Appraise/the social gradient	Judge what inequities exist in society in view of living a healthy life	5.2	23.2	50.9	19.0	1.7
Q16	Appraise/social exclusion	Judge what kind of government services should be supplied to those really in need of support	2.2	16.1	46.7	33.6	1.4
Q17	Appraise/work	Judge what level of burden of work has on health	2.9	22.1	52.2	21.7	1.1
Q18	Appraise/social support	Judge what kind of support should be supplied to someone in trouble in the community or workplace	1.7	15.3	52.7	29.6	0.7
Q19	Appraise/social capital	Judge how neighbors should help each other	3.1	18.2	53.7	24.2	0.8
Q20	Appraise/food	Judge the merits and demerits of the spread of processed foods	3.4	28.2	47.9	19.7	0.8
Q21	Appraise/transport	Judge the kind of impact that motorization has on health	4.2	29.8	50.5	14.1	1.3
Q22	Apply/social gradient	Cooperate in the creation of a fair society in which everyone can live a healthy life	3.7	18.2	47.1	29.0	2.0
Q23	Apply/early life	Involve oneself in politics and public administration to help small children live a healthy life	1.6	13.4	45.8	36.5	2.8
Q24	Apply/early life	Participate in childcare support activities	3.2	18.5	46.5	24.9	6.9
Q25	Apply/social exclusion	Participate in activities to eliminate poverty	1.4	11.8	46.7	36.7	3.4
Q26	Apply/work	Involve oneself in politics and public administration to protect the health of workers both institutionally and legally	1.4	10.3	43.6	42.4	2.3
Q27	Apply/work	Approach the manager or the employer regarding rewards that do not match efforts done at work	1.8	11.6	41.9	41.3	3.5
Q28	Apply/unemployment	Participate in activities to increase employment and vocational training opportunities	2.0	14.9	51.1	28.5	3.4
Q29	Apply/social support	Participate in activities that support an individual, including his or her family, who is in trouble in the community or workplace	2.2	13.5	50.2	32.1	2.0
Q30	Apply/social capital	Participate in activities to spread the importance of ties with people for health	2.6	19.3	50.9	26.4	0.8
Q31	Apply/addiction	Involve oneself in politics and public administration to make it easier for persons who have used illegal drugs to receive treatment	1.3	10.2	43.3	41.9	3.2
Q32	Apply/food	Participate in activities that promote a healthy diet	3.2	26.5	48.0	21.4	0.8
Q33	Apply/transport	Involve oneself in politics and public administration to seek road priority for pedestrians and cyclists	1.6	15.9	48.0	33.1	1.4

Regarding the total difficult (“very difficult” + “fairly difficult”), Q26 “Involve oneself in politics and public administration to protect the health of workers both institutionally and legally” (86.0%) is the most difficult item. Many items of the ability to apply information related to the social determinants are recognized as more difficult. By contrast, regarding the total easy (“very easy” + “fairly easy”), Q12 “Understand that work that is not stable becomes a huge stress” (66.1%) is the easiest item. Many items of to understand are recognized as easier.

### Reliability: internal consistency

The Cronbach’s alpha coefficient values were calculated to examine the internal consistency of the scale and its component subscales. For the questionnaire, Cronbach’s alpha was 0.92; the subscale values were 0.82 for accessing, 0.91 for understanding, 0.84 for appraising, and 0.92 for applying items. For all scales, the reliability of ≥0.8 was achieved and the item-total correlations were all positive.

### Construct validity

#### Factor structure

CFA was conducted to assess factorial validity, revealing CFI and RMSEA values of 0.901 and 0.058, respectively. Four-factor models for the questionnaire item fitted the data reasonably well.

#### Bivariate analysis

Bivariate relationships between the HL-SDHQ scores and other parameters are shown in Tables 4 and 5. Scores on the HL-SDHQ was significantly associated with the HP-HL (*r* = 0.74, *p* < 0.001), as well as health status (*r* = 0.14, *p* = 0.001), gender (*F* = 4.168, *p* = 0.042), and self-assessed living conditions (*F* = 2.932, *p* = 0.020) (Table 4).

**Table 4:** Bivariate relationships of HL-SDH scales with other measures

	HL-SDH			
Variables	Mean ± SD	r	F	*P*
HP-HL index of Japanese version HLS-EU-Q47		.74		<0.001
Self-rated health		.14		.001
Gender				
Men	68.8 ± 13.7		4.168	.042
Women	70.7 ± 13.6			
Age in categories				
20–29	69.8 ± 13.7		0.614	.653
30–39	70.1 ± 14.7			
40–49	68.8 ± 13.6			
50–59	68.8 ± 13.8			
60–69	70.7 ± 12.7			
Education attainment				
Junior high school	75.0 ± 12.0		1.645	.161
High school	69.6 ± 13.5			
Junior college	71.1 ± 14.1			
College/university	68.6 ± 13.9			
Graduate school	70.9 ± 12.6			
Annual pre-tax household income(million yen^a^)				
< 1.5	67.7 ± 12.5		1.909	.091
1.5–3.5	68.0 ± 14.2			
3.5–6.0	68.9 ± 13.3			
6.0–8.5	70.5 ± 14.7			
8.5–12.5	72.2 ± 14.4			
≥ 12.5	72.8 ± 12.1			
Self-assessed Living Conditions				
Very Hard	67.2 ± 16.8		2.932	.020
Hard	68.4 ± 12.9			
Common	70.1 ± 13.2			
A Little Well	71.2 ± 14.4			
Very well	78.0 ± 16.7			
Occupation				
Self-employed	71.8 ± 16.3		0.79	.596
Managerial and administrative	69.0 ± 13.8			
Professional and technical	68.9 ± 14.7			
Others (Routine and manual)	69.6 ± 14.7			
Part time	68.2 ± 12.2			
Homemaker	70.8 ± 12.8			
Student	73.1 ± 10.5			
Unemployed	68.6 ± 12.3			
Municipality size				
Very large(population of ≥500,000)	70.3 ± 14.2		1.985	.115
Large (population of 100,000–499,999)	70.0 ± 13.8			
Moderate (population of <100,000)	67.3 ± 12.9			
Small	70.9 ± 13.0			

^a^$1 = approximately 102 yen in Sep 2016

**Table 5:** Association of civic activity and HL-SDH and HP-HL

	HL-SDH		HP-HL	
	r	*P*	r	*P*
Support activities for mother and child related to natural childbirth and child rearing	.22	<.001	.11	.001
Disaster support and disaster prevention activities for supporting disaster-stricken areas and victims	.22	<.001	.16	<0.001
Group purchase of pesticide-free vegetables	.22	<.001	.18	<0.001
Activities for promoting folk entertainment, culture, and encouraging tourism	.22	<.001	.18	<0.001
Cooperative activities and consumer movements	.21	<.001	.14	<0.001
Environmental conservation and ecology movements such as recycling and prevention of pollution	.21	<.001	.14	<0.001
Activities to help the elderly and persons with disabilities	.20	<001	.16	<0.001
International cooperational activities for providing support to poor countries	.19	<.001	.12	<0.001
Political activities and support and recommendation of lawmakers	.19	<.001	.11	.002
Activities of administration volunteers of museums and art galleries	.19	<.001	.12	<0.001
Activities related to protection of human rights and elimination of discrimination	.18	<.001	.11	.001
Petitions to the administration	.18	<.001	.09	.009
Intercultural communication activities including international exchange	.18	<.001	.14	<0.001
Collection of waste materials or exchange of unwanted articles	.17	<.001	.13	<0.001
Activities involving mutual discussions about suffering and troubles	.17	<.001	.12	<0.001
Activities related to the development of children and youth	.17	<.001	.12	.001
Activities related to the safety of the community, such as fire prevention, disaster prevention, crime prevention, and traffic safety	.17	<.001	.14	<.001
Activities against the use of nuclear weapons and anti-nuclear power plants	.16	<.001	.10	.007
Activities promoting sports	.16	<.001	.16	<0.001
Activities with good health as the purpose such as meetings to think about health and medical care	.16	<.001	.16	<0.001
Environment beautification activities such as cleaning and weeding of roads and parks	.14	<.001	.13	<0.001
Activities of children and youth sports teams	.13	<.001	.10	.007
PTA gatherings and gatherings of parents of nurseries and kindergartens	.12	<.001	.08	.017
Athletics and various sports and recreational activities	.12	<.001	.13	<0.001
Bon dance and festivals	.11	.002	.11	.001

Bon dance is the festival folk dance in Japan

In addition, we also found significant relationships between the HL-SDHQ and the frequency of participation in citizens’ activities (Table 5). The results showed high correlations for “Support activities for mother and child related to natural childbirth and child rearing,” “Disaster support and disaster prevention activities for supporting disaster-stricken areas and victims,” and “Group purchase of pesticide-free vegetables” (*r* = 0.22 for each). Further, the correlation coefficient between these activities and the HL-SDHQ was higher than the corresponding value for the HP-HL (*r* = 0.11, 0.16, 0.18 respectively).

A significant correlation (*r* = 0.20; data not shown) was also obtained between the HL-SDHQ and the number of participation in citizens’ activities.

## Discussion

In this study, we developed a health literacy scale on social determinants of health. As described by Sorensen et al. [4, 11], when a citizen has high health literacy, he or she is striving for optimal health when encountering health promotion efforts in community, workplace, educational system, and marketplace. However, to date, health literacy related to the social determinants of health has not been adequately measured, and the importance of this type of health literacy has only been mentioned theoretically. With the HL-SDHQ, we expect that the importance of this type of health literacy can be clarified in future research.

HL-SDHQ showed high reliability with Cronbach’s alpha values ranging from 0.82 to 0.92. Construct validity was confirmed by a CFA model with the four factors of information-processing dimensions (accessing, understanding, appraising, and applying) and an adequate goodness of fit. In the bivariate relationship, a valid result was obtained for the relationships with the HP-HL, the frequency and the number of participation in citizens’ activities, and the self-rated health. The correlation coefficient between the HL-SDHQ and HP-HL was high at 0.74, indicating the validity of the HL-SDHQ to measure health literacy required for health promotion. Further, when we compared the Spearman’s correlation coefficient between the frequency of participation in citizens’ activities and the HL-SDHQ with the corresponding value for the HP-HL, the HL-SDHQ rather than HP-HL had a much strong relationship with activities such as “Support activities for mother and child related to natural childbirth and child rearing” or “Group purchase of pesticide-free vegetables” in which health was an objective that changed society. This result not only supports the theory critical health literacy proposed by Nutbeam [5] but also indicates the possibility of assessment of community empowerment using the HL-SDHQ.

Although the HL-SDHQ is different from health literacy scale already developed because it does not focus on the realization of the health of a specific individual, but is instead directed toward the realization of the health of the community or society through modifications to the social determinants of health, a positive correlation was obtained for the relationship between HL-SDHQ and self-rated health. Generally, similar results are obtained from studies reporting health literacy and self-rated health [30]. According to the WHO [33], to reach a state of complete physical, mental and social well-being, an individual or group must be able to identify and realize aspirations, satisfy needs, and change or cope with the environment. Health is therefore seen as a resource for everyday life, not merely the objective of living, and is a positive concept emphasizing social and personal resources, as well as physical capacities. In short, if a person has a high score on the HL-SDHQ, there is a higher possibility of increased well-being, making the relationship with the self-assessment of current health valid. However, in a report by Nakayama et al., the correlation coefficient between the self-rated health and the HLS-EU-Q47 was 0.18, compared with the lower value of 0.14 for the HL-SDHQ. This may be because the HLS-EU-Q47 includes topics that directly impact the individual’s health, including illnesses and lifestyle habits, while the HL-SDHQ included only those items considered social determinants of health, or “cause of cause.”

In the relationship with basic attributes, no relationship was seen with objective indicators of social position, such as education or income, but there was a significant relationship with subjective life circumstances. This result is similar to the results of the HLS-EU-Survey in the Netherlands [34]. An important topic for future research would be to examine the influence on the HL-SDHQ of indicators of subjective life circumstances rather than objective indicators of social position.

Although no significant relationship was observed with age, it is important to note that the HL-SDHQ score was high for those aged over 60 years. In a previous study, it was conceivable that because cognitive ability may decline when one becomes older, health literacy could also decline; however, the results of this study do not support this argument [35]. The reason for the high HL-SDHQ score for those aged 60 years and older could be attributed to the fact that those with consciousness of the need for social contribution will participate in community activities when they retire in Japan. Perhaps because of this, the number of people contributing to building a better society increases. Concerning the relationship between gender and health literacy, we found a significantly higher score for females; however, previous research has shown that there is no consistent relationship between gender and health literacy [34].

It is important to note that this study has some limitations. First, it is possible that there was some selection bias. The Japanese cohort may have been skewed toward including those with a high level of internet literacy because of the use of a web-based survey. Also, the recruitment of respondents was based on self-selection from among a group of individuals who had previously expressed a desire to participate in research projects. Moreover, responses were limited to the first 1000 people, and may therefore have only included those who were most active on the internet. When we confirmed the representativeness of the study sample to the general population in terms of age, gender, income and educational level, participants included a more men and a higher education level. Perhaps, these factors might positively influence internet literacy. This suggests that the HL-SDHQ may be validated for web-based studies.

Second, our findings may be limited to Japanese settings, and care must be exercised especially when generalizing them to European and American countries. Given that the development and research for the HL-SDHQ has been implemented in Japanese, the validity and reliability of an English version need to be assessed in future research.

## Conclusion

In this study, we developed a reliable and valid health literacy scale on the social determinants of health in Japan. We operationally identified ten domains of the social determinants of health and confirmed their content validity. Moreover, a high goodness of fit was obtained and we showed a clear bivariate relationship between the HL-SDHQ and the frequency and the number of participation in citizens’ activities, supporting the validity. In the future, there is a need to confirm the usefulness of each question and to confirm the validity of the questionnaire in other cultural settings and languages.

## Additional file

Additional file 1: HL-SDHQ original version (Japanese version) [see Additional file 1]. (DOCX 23 kb)

## Abbreviations

CFA: Confirmatory factor analysis
CFI: Comparative fit index
CSDH: Commission on the Social Determinants of Health
HL-SDHQ: Health Literacy on Social Determinants of Health Questionnaire
HP-HL: Health promotion health literacy index
RMSEA: Root mean square error of approximation
WHO: World Health Organization
