# Supplementary material for: Development of the health literacy on social determinants of health questionnaire in Japanese adults
Source: BMC Public Health. 2017 Jan 6;17:30. doi: 10.1186/s12889-016-3971-3 (PMC5217562; doi:10.1186/s12889-016-3971-3)
Supplement: Additional file 1: — HL-SDHQ original version (Japanese version) [see Additional file 1]. (DOCX 23 kb) [file 12889_2016_3971_MOESM1_ESM.docx]

HL-SDHQ Original version (日本語版)

| 以下のそれぞれが、あなたにとって簡単か難しいかについてお聞きします。それぞれ「とても簡単」から「とても難しい」までで、最もあてはまるものに○を付けてください（それぞれひとつずつ）。 | | とても簡単 | やや 簡単 | やや 難しい | とても難しい | わからない/あてはまらない |
| --- | --- | --- | --- | --- | --- | --- |
| (1) | 社会的な地位が健康に影響を与えることについて知るのは | 1 | 2 | 3 | 4 | 5 |
| (2) | 妊娠中の母親の生活が、生まれる子供の成長に与える影響に関する情報を見つけるのは | 1 | 2 | 3 | 4 | 5 |
| (3) | 社会から孤立して健康を損ねている人を見つけるのは | 1 | 2 | 3 | 4 | 5 |
| (4) | 労働者の失業とストレスの関係に関する情報を見つけるのは | 1 | 2 | 3 | 4 | 5 |
| (5) | 地域や職場で困っている人が必要な支援について知るのは | 1 | 2 | 3 | 4 | 5 |
| (6) | 喫煙がストレスの原因の解決にならないことについて知るのは | 1 | 2 | 3 | 4 | 5 |
| (7) | 食生活の変化と健康の関係に関する情報を見つけるのは | 1 | 2 | 3 | 4 | 5 |
| (8) | 所得の少ない人ほど、病気になりがちであると理解するのは | 1 | 2 | 3 | 4 | 5 |
| (9) | 子供の頃に受けた虐待は、大人になっても影響すると理解するのは | 1 | 2 | 3 | 4 | 5 |
| (10) | 地域や職場で孤立していることは、健康に影響すると理解するのは | 1 | 2 | 3 | 4 | 5 |
| (11) | 仕事の進め方を自分で決められることは、ストレスと関連すると理解するのは | 1 | 2 | 3 | 4 | 5 |
| (12) | 雇用が安定しない仕事は、大きなストレスになると理解するのは | 1 | 2 | 3 | 4 | 5 |
| (13) | 所得格差の拡大は、人々のつながりを希薄にすると理解するのは | 1 | 2 | 3 | 4 | 5 |
| (14) | ストレスの多い社会では、薬物への依存が起こりやすいと理解するのは | 1 | 2 | 3 | 4 | 5 |
| (15) | 社会には、健康な生活を送るうえでどのような不公平があるかを判断するのは | 1 | 2 | 3 | 4 | 5 |
| (16) | 支援が本当に必要な人に、どのような行政サービスが提供されるべきかを判断するのは | 1 | 2 | 3 | 4 | 5 |
| (17) | 仕事の負担感は、どの程度あると健康に影響するかを判断するのは | 1 | 2 | 3 | 4 | 5 |
| (18) | 地域や職場で困っている人に、どのような支援を提供すべきかを判断するのは | 1 | 2 | 3 | 4 | 5 |
| (19) | ご近所同士は、どのように助け合っていけばよいかを判断するのは | 1 | 2 | 3 | 4 | 5 |
| (20) | 加工食品の普及による長所と短所を判断するのは | 1 | 2 | 3 | 4 | 5 |
| (21) | 車社会は健康にどのような影響を与えるかを判断するのは | 1 | 2 | 3 | 4 | 5 |
| (22) | 誰もが健康でいられる公平な社会をつくるために協力するのは | 1 | 2 | 3 | 4 | 5 |
| (23) | 小さい子供が健康に暮らせるように、政治や行政に働きかけるのは | 1 | 2 | 3 | 4 | 5 |
| (24) | 育児支援を行っている活動に参加するのは | 1 | 2 | 3 | 4 | 5 |
| (25) | 貧困をなくすための活動に参加するのは | 1 | 2 | 3 | 4 | 5 |
| (26) | 労働者の健康を守るための制度や法律を求めて、政治や行政に働きかけるのは | 1 | 2 | 3 | 4 | 5 |
| (27) | 仕事上の努力に見合わない報酬に対して、上司や雇用者に働きかけるのは | 1 | 2 | 3 | 4 | 5 |
| (28) | 就職や職業訓練の機会を増やすための活動に参加するのは | 1 | 2 | 3 | 4 | 5 |
| (29) | 地域や職場で困っている人やその家族を支援するための活動に参加するのは | 1 | 2 | 3 | 4 | 5 |
| (30) | 健康のために、人とのつながりが大切なことを広める活動に参加するのは | 1 | 2 | 3 | 4 | 5 |
| (31) | 不法薬物を使用した人が治療を受けやすくなるように、政治や行政に働きかけるのは | 1 | 2 | 3 | 4 | 5 |
| (32) | 健康的な食事を推進するための活動に参加するのは | 1 | 2 | 3 | 4 | 5 |
| (33) | 歩行者や自転車利用者が優先される道路を求めて、政治や行政に働きかけるのは | 1 | 2 | 3 | 4 | 5 |

作成代表者　松本真欣（ユニバーサル・ビジネス・ソリューションズ）masayoshi.matsumoto@ubsc.co.jp
